# Sleep and its Association With Pain and Depression in Nursing Home Patients With Advanced Dementia – a Cross-Sectional Study

**DOI:** 10.3389/fpsyg.2021.633959

**Published:** 2021-04-20

**Authors:** Kjersti Marie Blytt, Elisabeth Flo-Groeneboom, Ane Erdal, Bjørn Bjorvatn, Bettina S. Husebo

**Affiliations:** ^1^Department of Health and Caring Sciences, Western Norway University of Applied Sciences, Bergen, Norway; ^2^Faculty of Psychology, University of Bergen, Bergen, Norway; ^3^Norwegian Competence Center for Sleep Disorders, Haukeland University Hospital, Bergen, Norway; ^4^Department of Global Public Health and Primary Care, University of Bergen, Bergen, Norway; ^5^Centre for Elderly and Nursing Home Medicine, University of Bergen, Bergen, Norway; ^6^Municipality of Bergen, Norway

**Keywords:** sleep, pain, depression, nursing home patients, actigraphy, dementia

## Abstract

**Objective:** Previous research suggests a positive association between pain, depression and sleep. In this study, we investigate how sleep correlates with varying levels of pain and depression in nursing home (NH) patients with dementia.

**Materials and methods:** Cross-sectional study (*n* = 141) with sleep-related data, derived from two multicenter studies conducted in Norway. We included NH patients with dementia according to the *Mini-Mental State Examination* (MMSE ≤ 20) from the COSMOS trial (*n* = 46) and the DEP.PAIN.DEM trial (*n* = 95) whose sleep was objectively measured with actigraphy. In the COSMOS trial, NH patients were included if they were ≥65 years of age and with life expectancy >6 months. In the DEP.PAIN.DEM trial, patients were included if they were ≥60 years and if they had depression according to the *Cornell Scale for Depression in Dementia* (CSDD ≥ 8). In both studies, pain was assessed with the Mobilization-Observation-Behavior-Intensity-Dementia-2 Pain Scale (MOBID-2), and depression with CSDD. Sleep parameters were total sleep time (TST), sleep efficiency (SE), sleep onset latency (SOL), wake after sleep onset (WASO), early morning awakening (EMA), daytime total sleep time (DTS) and time in bed (TiB). We registered use of sedatives, analgesics, opioids and antidepressants from patient health records and adjusted for these medications in the analyses.

**Results:** Mean age was 86.2 years and 76.3% were female. Hierarchical regressions showed that pain was associated with higher TST and SE (*p* < 0.05), less WASO (*p* < 0.01) and more DTS (*p* < 0.01). More severe dementia was associated with more WASO (*p* < 0.05) and TiB (*p* < 0.01). More severe depression was associated with less TST (*p* < 0.05), less DTS (*p* < 0.01) and less TiB (*p* < 0.01). Use of sedative medications was associated with less TiB (*p* < 0.05).

**Conclusion:** When sleep was measured with actigraphy, NH patients with dementia and pain slept more than patients without pain, in terms of higher total sleep time. Furthermore, their sleep efficiency was higher, indicating that the patients had more sleep within the time they spent in bed. Patients with more severe dementia spent more time awake during the time spent in bed. Furthermore, people with more severe depression slept less at daytime and had less total sleep time Controlling for concomitant medication use did not affect the obtained results.

## Introduction

Approximately 50 million people are living with dementia worldwide, and every year 10 million new cases are registered (World Health Organization [WHO], 2019). Sleep disturbances, like difficulties in initiating sleep, frequent awakenings, excessive daytime sleepiness and nighttime wandering are often seen in people with dementia (McCleery et al., 2016; Webster et al., 2020a). However, to estimate the prevalence of sleep disturbances in people with dementia is difficult due to differences in assessment methods and because of the patients’ reduced communicative ability (Blytt et al., 2017).

A recent systematic review and meta-analysis conducted by Webster et al. (2020b) found a 20% pooled prevalence of clinically significant sleep disturbances in people with dementia and a 70% prevalence of sleep disturbances as measured with actigraphy. Furthermore, Webster et al. (2020a) found that sleep disturbances negatively affect physical and psychological well-being in people with dementia. In the NH setting, sleep disturbances also caused disruption for other patients and increased stress among staff (Webster et al., 2020a). Previously conducted studies have found that sleep disturbances in dementia are associated with reduced quality of life and impaired daytime functioning (Kyle et al., 2010), increased family burden and earlier nursing home (NH) admission, thereby increasing the social and economic costs for society (McCrae et al., 2016; Livingston et al., 2017).

Pain is a subjective experience that is difficult to assess for people with dementia, due to reduced capacity to give valid self-report (Blytt et al., 2017). When assessed by proxy-rater measurements, moderate to severe pain is found in approximately 40–60% of NH patients with dementia (Achterberg et al., 2010; Hemmingsson et al., 2018; Wagatsuma et al., 2020). Reduced quality of life, increased agitation and anxiety are all associated with pain in NH patients (Ballard et al., 2009). In addition, approximately 50% of people with dementia experience symptoms of depression – a condition that may reduce their quality of life and accelerate the decline in cognition and daily functioning (Enache et al., 2011).

Previous research has found a reciprocal relationship between pain and depression in people without dementia (Bair et al., 2003; Goldenberg, 2010; Kroenke et al., 2011). This relationship is known as the “pain-depression dyad,” indicating that the conditions often coexist, respond to similar treatment and share common signal pathways (Chopra and Arora, 2014). In NH patients, Erdal et al. (2017) found that reduced pain intensity was associated with subsequent reduction of depressive symptoms, regardless of analgesic or antidepressant use. These associations were found in patients with mild, moderate and severe cognitive impairment. However, results on the primary outcome measures

in an analgesic intervention trial did not support a direct causal relationship between pain and increased depression in NH patients with dementia (Erdal et al., 2018).

There is also considerable evidence for a reciprocal relationship between pain and sleep in people without dementia. Previous research suggests that poor sleep may affect the perception of pain (Sivertsen et al., 2015), but also that pain in itself can be a possible cause of poor sleep (Sivertsen et al., 2015; Flo et al., 2017). There is some evidence suggesting that depression may be a central underlying mechanism in the relationship between pain and sleep in people without cognitive impairment (Ravyts et al., 2018). Furthermore, pain may lead to depression, which in turn may affect the patient’s sleep (Nicassio et al., 2012; Flo et al., 2017). By extension people with dementia and depression may be lying in bed, experiencing trouble falling asleep or maintaining sleep because they are in pain (Blytt, 2019). However, there is still a lack of evidence regarding this relationship in people with dementia (Flo et al., 2017).

To our knowledge, there are no previous studies investigating how sleep is associated with pain and depression in NH patients with dementia. Given the frequency and clinical implications of sleep disturbances for the patients, co-residents and staff, new knowledge regarding the relationship between pain, depression and sleep in this patient group is valuable. Consequently, we aimed to investigate the association between sleep, pain, depression and dementia in NH patients. We hypothesized that a) more pain was associated with poorer sleep; b) more severe dementia was associated with poorer sleep; and c) more severe depression was associated with poorer sleep. Because medical treatment for pain, depression and sleep disorders may also impact sleep parameters, analyses were adjusted for the use of analgesic, antidepressant and sedative-hypnotic drugs.

## Materials and Methods

### Participants and Protocol

The study was based on sleep-related data derived from two independent multicenter studies conducted in Norway: the COSMOS trial (*n* = 46) and the DEP.PAIN.DEM trial (*n* = 95). The COSMOS (*Communication, Systematic pain assessment and treatment, Medication review, Organization of activities, and Safety*) trial is a multicenter cluster-randomized controlled trial over 4 months with follow-up at month 9. The study was conducted from January 2014 to December 2015. It aimed to improve the quality of life among NH patients through a multicomponent intervention (Husebo et al., 2015). A total of 765 patients were invited to participate in the COSMOS study, and 545 participants from 67 NH units were included (Husebø et al., 2019). The COSMOS trial used the following inclusion and exclusion criteria: NH patients ≥65 years of age and life expectancy >6 months.

The DEP.PAIN.DEM trial is a multicenter, placebo-controlled randomized clinical trial over 13 weeks. The study was conducted in Norway from August 2014 to September 2016. The main aim of the DEP.PAIN.DEM trial was to investigate the efficacy of pain treatment on depression in people with dementia and depression. Patients were included if they were long-term NH patients ≥60 years with >4 week stay at inclusion, dementia according to the Mini-Mental State Examination (MMSE score ≤ 20) and depression according to the Cornell Scale for Depression in Dementia (CSDD score ≥ 8). The sleep subproject of DEP.PAIN.DEM included 106 patients from 47 NHs (Blytt et al., 2018a).

Patients with Parkinson disorder or any form of paralysis in the arms or upper body were excluded from actigraphy registrations. In the present study, we included patients who had valid actigraphy measurements and dementia according to MMSE (≤20) from both the DEP.PAIN.DEM and the COSMOS trials, which implies that we used data collected at baseline (week 0) before any intervention was conducted.

In total, 141 patients from the COSMOS (*n* = 46) and the DEP.PAIN.DEM (*n* = 95) trials had valid actigraphy measurements and dementia according to MMSE and were included in the present study. Some of the respondents did not have complete datasets for all variables (see Table 1 for the number of observations for the different variables). Mean age was 86.2 years and 76.3% were female.

**TABLE 1:** Descriptive statistics: age, gender, CSDD, MOBID-2, and MMSE scores for the full sample.

	Descriptives	No. of observations
Age (mean, SD)	86.2 (7.5)	140
Female (%)	76.3	141
CSDD (mean, SD)	9.5 (4.7)	134
MOBID-2 (mean, SD)	2.6 (2.3)	120
MMSE (mean, SD)	8.8 (5.8)	141

*Descriptive statistics for the full sample (*n* = 141). Number of observations refers to how many patients have valid data for the different parameters. Abbreviations: CSDD = Cornell Scale for Depression in Dementia, MOBID-2 = Mobilization-Observation-Behavior-Intensity-Dementia-2 Pain Scale, MMSE = Mini-Mental State Examination).*

In the data collection process, the patient’s medical decision-making capacity was discussed with the patient’s primary nurse at the NH. Efforts were made to adjust the information for patients who had reduced capacity to give consent (MMSE score from 16 to 19). In addition, the researchers telephoned all the eligible patients’ legal guardians. If the legal guardians gave presumed consent on behalf of the patient, they received written and oral information together with a consent form that they signed and mailed back. The DEP.PAIN.DEM study was approved by the Regional Ethics Committee (REC-West 2013/1474). The study’s clinical trial number is NCT02267057. The COSMOS trial was also approved by the Regional Committee for Medical and Health Research Ethics, West Norway (REK 2013/1765) and registered at www.clinicaltrials.gov (NCT02238652).

### Measurements

In both studies, sleep was measured with *Actiwatch Spectrum (Philips Respironics)* for seven consecutive days.

NH patients may be quite inactive and in order to enhance the possibility that movement would be detected we placed the actigraphs on the patients’ dominant wrist. Prior studies have found no discrepancy between data gathered from actigraphs placed on different locations (Sadeh et al., 1994; Jean-Louis et al., 1997). To enable better scoring of the actual time patients spent in bed, NH staff was instructed to register bedtimes and rise times by pushing the event button on the actigraph (light off at night and light on in the morning). Instructions were given both verbally and on a written instruction that was placed above the nightstand in the patients’ rooms and in the call room.

The *Actiware 6 (Respironics)* scoring program and validated algorithm was used for sleep/wake scoring. An actigraph consists of an accelerometer and memory storage (Sivertsen et al., 2006). Based on differences in movements associated with wakefulness and sleep, an estimate of sleep-wake schedules is provided (Sivertsen et al., 2006). We used medium sensitivity since this is most commonly used., and sleep/waking status was determined for each one-minute epoch. A skilled technician scored all activity protocols. A standardized hierarchical approach was applied to set rest intervals for the actigraphy data, using a) event markers when possible; or b) light and activity data; or c) light or activity data. If there was clear differentiation between active and rest periods, alternatives b) and c) were implemented. If there was no such clear differentiation, the actigraphy protocol was excluded. In the main part of the DEP.PAIN.DEM study, 162 patients were enrolled (Erdal et al., 2018). However, 7 patients had Parkinson’s disease, 11 did not consent to wear an actigraph, 8 patients removed the actigraph and 30 were excluded for other reasons (missing data, malfunctioning actigraphs, etc.) and were thus excluded in the actigraph sub-project. As a result, 106 were included in the actigraph subproject in the DEP.PAIN.DEM -study. 95 of these patients had valid MMSE measurements and were thus included in the present study. In the COSMOS study the actigraphy subproject included 107 patients, 24 of whom were excluded due to actigraph malfunction or because of missing data. Of these 46 had dementia according to the MMSE measurements and were thus included in the final sample. The following parameters were derived from actigraphy measurements: total sleep time (TST, min), time in bed (TiB, hrs:min), sleep efficiency (SE,% [TST/TiB]^∗^100), sleep onset latency (SOL, min), wake after sleep onset (WASO, min), early morning awakening (EMA, min) and day time total sleep time (DTS, min). DTS is a measure of sleep during daytime (from light on in the morning to lights off at night) and TST is a measure of sleep during nighttime (from light off in the night to lights on in the morning).

We used the validated CSDD to measure depression (Barca et al., 2010). The CSDD consists of 19 items assessing five domains of depression (mood, behavioral disturbances, physical signs, cyclic functions and ideational disturbances). A cut-off point of 8/9 has demonstrated the best accuracy for diagnosing depression, ≥11 corresponds with severe depression according to the ICD-10 criteria (Barca et al., 2010). We used the following cut-offs in classifying the categorical variables: no depression = CSDD ≤ 6, moderate depression = 7 ≤ CSDD ≤ 10, severe depression = CSDD ≥ 11 (Barca et al., 2010).

Cognitive function was assessed using the validated MMSE (Perneczky et al., 2006). The MMSE is a cognitive screening test with a 30-point scale consisting of 20 tasks, which was developed to differentiate potential dementia from normal functioning. In the present study, we used the following cut-offs: a score from 0 to 10 represents severe dementia, a score from 11 to 20 represents moderate dementia, a score from 21 to 25 represents mild cognitive impairment, and a score from 26 to 30 represents no dementia (Perneczky et al., 2006).

To measure pain, we used the Mobilization-Observation-Behavior-Intensity-Dementia-2 Pain Scale (MOBID-2), a validated staff-administered instrument (Husebo et al., 2010). The instrument provides a total score based on observations ranging from 0 to 10, in which 10 represents the worst possible pain. In line with previous research, a cut-off of ≥3 was used to indicate clinically relevant pain (Husebo et al., 2007, 2014).

Use of the following medications was registered based on daily prescriptions registered in the patient health records: N02A (opioids), N02B (other analgesics), N05C (hypnotics and sedatives) and N06A (antidepressants). Patients were registered categorically as users or non-users within each ATC group.

### Statistical Analyses

Descriptive statistics (means, standard deviations and percentages) were calculated for the full sample (Table 1) and for the DEP.PAIN.DEM dataset and the COSMOS dataset separately (Table 2). In addition, we conducted independent samples *t*-tests to investigate if there were any statistically significant differences in relevant characteristics between the respondents in the two studies. Sleep characteristics were calculated for the full sample, for patients with and without pain as measured with MOBID-2, for patients with different degrees of depression according to the CSDD, and with different degrees of cognitive function according to the MMSE. We also conducted independent samples *t*-tests to investigate if there were any statistically differences between any of the different sleep parameters regarding these categories (pain/no pain, moderate dementia/severe dementia and no depression/moderate depression versus severe depression, Table 3).

**TABLE 2:** Descriptive statistics for age and CSDD, MOBID-2, and MMSE scores, comparing data from the DEP.PAIN.DEM and COSMOS studies.

	DEP.PAIN.DEM (*n* = 95)	COSMOS (*n* = 46)	*P*
Age (mean,SD)	85.6 (7.5)	87.6 (7.6)	0.13
CSDD (mean, SD)	11.1 (3.4)	5.5 (5.4)	0.00
MOBID-2 (mean, SD)	2.8 (2.2)	2.3 (2.4)	0.25
MMSE (mean, SD)	7.6 (6.0)	11.2 (4.5)	0.00

*Descriptive statistics for the DEP.PAIN.DEM dataset and the COSMOS dataset. Independent samples *t*-test show statistically significant differences between the CSDD and MMSE scores.*

**TABLE 3:** Panel A: Sleep outcomes for the full sample, *n* = 141 (mean, SD)

TST (min)	516.3 (127.8)
SE (%)	70.2 (14.9)
SOL (min)	39.4 (51.6)
WASO (min)	138.8 (71.6)
EMA (min)	39.3 (44.3)
DTS (min)	210.3 (116.3)
TiB (hrs:min)	12:14 (1:14)

*The following abbreviations are used: TST = total sleep time, SE = sleep efficiency, SOL = sleep onset latency, WASO = wake after sleep onset, EMA = early morning awakening, DTS = daytime total sleep time, TiB = time in bed. The same abbreviations apply for all panels below.*

**TABLE 3:** Panel B: Sleep outcomes for patients with/without pain, *n* = 121 (mean, SD)

	No pain (*n* = 61)	Pain (*n* = 60)	*p*
TST (min)	489.0 (101.0)	542.9 (148.0)	**0.046**
SE (%)	67.6 (13.1)	73.2 (16.8)	**0.025**
SOL (min)	37.5 (41.6)	37.7 (61.5)	0.435
WASO (min)	156.1 (76.1)	123.3 (65.0)	**0.020**
EMA (min)	43.5 (40.2)	33.5 (42.0)	0.190
DTS (min)	181.3 (98.4)	239.8 (131.6)	**0.003**
TiB (hrs:min)	12:07 (1:16)	12:17 (1:10)	0.916

**TABLE 3:** Panel C: Sleep outcomes for patients with degrees of depression, *n* = 133 (mean, SD)

	No depression (*n* = 26)	Moderate depression (*n* = 55)	Severe depression (*n* = 52)	*p*
TST (min)	548.5 (113.5)	521.9 (142.8)	497.7 (119.0)	0.985
SE (%)	72.9 (14.6)	71.5 (16.7)	67.9 (13.0)	0.830
SOL (min)	35.7 (47.5)	39.1 (62.1)	43.2 (44.2)	0.555
WASO (min)	138.3 (90.7)	127.5 (63.8)	150.1 (70.1)	0.281
EMA (min)	33.4 (32.3)	37.9 (48.2)	39.9 (42.8)	0.446
DTS (min)	265.5 (118.0)	211.0 (116.0)	193.4 (109.7)	0.343
TiB (hrs:min)	12:35 (1:05)	12:07 (1:08)	12:11 (1:25)	0.417

*No depression = CSDD < 6, moderate depression = 7 < CSDD < 10, severe depression = CSDD ≥ 11.*

**TABLE 3:** Panel D: Sleep outcomes for patients with different levels of dementia, *n* = 141 (mean, SD).

	Moderate dementia (*n* = 64)	Severe dementia (*n* = 77)	*p*
TST (min)	516.0 (113.6)	516.6 (139.2)	0.980
SE (%)	73.0 (11.9)	67.9 (16.8)	**0.039**
SOL (min)	32.0 (33.7)	45.6 (62.3)	0.101
WASO (min)	121.9 (50.3)	152.8 (83.1)	**0.007**
EMA (min)	34.5 (35.8)	43.4 (50.1)	0.224
DTS (min)	200.8 (114.5)	218.3 (117.9)	0.376
TiB (hrs:min)	11:45 (1:14)	12:38 (1:05)	**0.000**

*No dementia = MMSE ≥ 26, Mild dementia = MMSE score from 21 ≤ MMSE ≤ 25 to ≥ 25, Moderate dementia = MMSE score from 11 ≤ MMSE ≤ 20, Severe dementia = MMSE score MMSE ≤ 10.*

Two-step hierarchical multiple regression were conducted to investigate how the different sleep parameters (total sleep time, sleep efficiency, sleep onset latency, wake after sleep onset, early morning awakening, time in bed and daytime total sleep time) correlated with varying levels of pain, depression and dementia in NH patients (see Table 4). Multicollinearity tests using variance inflation factor showed no severe correlation between independent variables that warrant corrective measures These variables were entered in the first step of the hierarchical regressions. In the second step, we adjusted for the use of analgesics, antidepressants, sedative-hypnotics and opioids. These variables were implemented in the regression models to investigate their potential effect on the associations between the main variables of interest. We used categorical variables with the cut-off points described above for pain, depression and dementia. In addition, we include age and gender in both steps. We regarded *p* < 0.05 as statistically significant in all analyses.

**TABLE 4:** hierarchical regression models for various sleep parameters, with and without adjusting for medication use.

Panel A: Hierarchical regression models predicting total sleep time (TST) with and without adjusting for medication use.									

		Unstandardized coefficients		Standardized coefficients					
Step	Predictor	B	SE	β	p	R^2^	R^2^ change	F	p
1						0.095	0.095	2.24	0.056
	Pain	63.017	24.286	0.243	**0.011**				
	Depression	–36.388	16.941	–0.209	**0.034**				
	Dementia	8.307	25.043	0.032	0.741				
	Age	0.347	1.622	0.021	0.831				
	Gender	22.425	29.268	0.074	0.445				
2						0.147	0.052	1.96	0.052
	Pain	60.038	24.207	0.232	**0.015**				
	Depression	–34.612	17.183	–0.198	**0.047**				
	Dementia	3.150	25,756	0.012	0.903				
	Age	0.649	1.613	0.039	0.688				
	Gender	14.843	29.346	0.049	0.614				
	Analgesics	–0.435	24.880	–0.002	0.986				
	Sedatives	–55.265	29.105	–0.187	0.060				
	Antidepressants	22.888	24.473	0.088	0.352				
	Opioids	53.581	32.651	0.158	0.104				

*The table reports hierarchical regression models. Step 1 reports the correlates pain, depression, dementia, age, and gender. Step 2 also adjusts for analgesics, sedatives, antidepressants, and opioids. For unstandardized coefficients, B and SE signifies beta values and standard errors. For standardized coefficients, β and p signifies beta values and *p* values, respectively. F signifies the *F* value from statistical tests and *p* value associated with the test. The same information applies to all other panels below. Bold values indicate p-values < 0.05.*

**TABLE 4:** Panel B: Hierarchical regression models predicting sleep efficiency (SE) with and without adjusting for medication use.

		Unstandardized coefficients		Standardized coefficients					
Step	Predictor	B	SE	B	p	R^2^	R^2^ change	F	p
1						0.098	0.098	2.296	0.050
	Pain	6.706	2.821	0.223	**0.019**				
	Depression	–2.388	1.968	–0.118	0.228				
	Dementia	–5.299	2.909	–0.174	0.071				
	Age	–0.011	0.188	–0.006	0.954				
	Gender	3.503	3.400	0.099	0.305				
2						0.117	0.020	1.507	0.155
	Pain	6.438	2.865	0.214	**0.027**				
	Depression	–2.262	2.034	–0.111	0.268				
	Dementia	–5.484	3.048	–0.181	0.075				
	Age	0.005	0.191	0.003	0.977				
	Gender	2.933	3.473	0.083	0.400				
	Analgesics	0.793	2.944	0.026	0.788				
	Sedatives	–3.150	3.444	–0.092	0.363				
	Antidepressants	1.189	2.896	0.039	0.682				
	Opioids	4.852	3.864	0.123	0.212				

*Bold values indicate p-values < 0.05.*

**TABLE 4:** Panel C: Hierarchical regression models predicting sleep onset latency (SOL) with and without adjusting for medication use.

		Unstandardized coefficients		Standardized coefficients					
Step	Predictor	B	SE	β	p	R^2^	R^2^ change	F	P
1						0.024	0.024	0.520	0.760
	Pain	–2.236	10.225	–0.022	0.820				
	Depression	–1.881	7.132	–0.027	0.793				
	Dementia	15.290	10.543	0.145	0.150				
	Age	–0.209	0.683	–0.031	0.761				
	Gender	–6.753	12.322	–0.055	0.585				
2						0.046	0.022	0.552	0.833
	Pain	–1.950	10.375	–0.019	0.851				
	Depression	–1.369	7.365	–0.019	0.853				
	Dementia	18.519	11.039	0.175	0.096				
	Age	–0.284	0.691	–0.042	0.682				
	Gender	–4.465	12.578	–0.036	0.723				
	Analgesics	–8.861	10.664	–0.084	0.408				
	Sedatives	13.652	12.474	0.114	0.276				
	Antidepressants	–7.538	10.489	–0.072	0.474				
	Opioids	–6.741	13.994	–0.049	0.631				

**TABLE 4:** Panel D: Hierarchical regression models predicting wake after sleep onset (WASO) with and without adjusting for medication use.

		Unstandardized coefficients		Standardized coefficients					
Step	Predictor	B	SE	β	p	R^2^	R^2^ change	F	p
1						0.127	0.127	3.093	0.012
	Pain	–37.192	13.432	–0.255	**0.007**				
	Depression	6.513	9.370	0.066	0.488				
	Dementia	36.154	13.851	0.246	**0.010**				
	Age	0.854	0.897	0.091	0.343				
	Gender	–8.130	16.188	–0.048	0.617				
2						0.152	0.025	2.038	0.042
	Pain	–36.010	13.590	–0.247	**0.009**				
	Depression	5.077	9.647	0.052	0.600				
	Dementia	31.717	14.459	0.216	**0.031**				
	Age	0.907	0.905	0.096	0.319				
	Gender	–7.616	16.475	–0.045	0.645				
	Analgesics	–0.880	13.968	–0.006	0.950				
	Sedatives	–6.878	16.339	–0.041	0.675				
	Antidepressants	8.648	13.739	0.059	0.530				
	Opioids	–24.746	18.330	–0.130	0.180				

*Bold values indicate p-values < 0.05.*

**TABLE 4:** Panel E: Hierarchical regression models predicting early morning awakening (EMA) with and without adjusting for medication use.

		Unstandardized coefficients		Standardized coefficients					
Step	Predictor	B	SE	β	p	R^2^	R^2^ change	F	P
1						0.069	0.069	1.560	0.178
	Pain	–11.767	7.387	–0.152	0.114				
	Depression	4.197	5.153	0.080	0.417				
	Dementia	8.122	7.618	0.104	0.289				
	Age	–0.189	0.493	–0.038	0.703				
	Gender	–14.590	8.903	–0.161	0.104				
2						0.075	0.007	0.923	0.508
	Pain	–11.959	7.556	–0.154	0.117				
	Depression	3.926	5.364	0.075	0.466				
	Dementia	9.337	8.040	0.119	0.248				
	Age	0.228	0.503	–0.045	0.652				
	Gender	–13.902	9.160	–0.153	0.132				
	Analgesics	0.981	7.766	0.013	0.900				
	Sedatives	6.976	9.085	0.079	0.444				
	Antidepressants	–0.1022	7.639	–0.013	0.894				
	Opioids	0.289	10.192	0.003	0.977				

**TABLE 4:** Panel F: Hierarchical regression models predicting daytime total sleep time (DTS) with and without adjusting for medication use.

		Unstandardized coefficients		Standardized coefficients					
Step	Predictor	B	SE	β	p	R^2^	R^2^ change	F	P
1						0.169	0.169	4.261	0.001
	Pain	69.229	21.608	0.289	**0.002**				
	Depression	–49.988	15.272	–0.308	**0.001**				
	Dementia	31.684	22.409	0.131	0.160				
	Age	–0.311	1.455	–0.020	0.831				
	Gender	–31.897	26.025	–0.114	0.223				
2						0.205	0.036	2.891	0.004
	Pain	71.687	21.766	0.299	**0.001**				
	Depression	–44.710	15.652	–0.275	**0.005**				
	Dementia	25.192	23.223	0.105	0.281				
	Age	–0.052	1.457	–0.003	0.972				
	Gender	–39.097	26.257	–0.140	0.140				
	Analgesics	–0.492	22.312	–0.002	0.982				
	Sedatives	–51.510	26.053	–0.189	0.051				
	Antidepressants	–19.086	22.008	–0.080	0.390				
	Opioids	12.829	29.960	0.040	0.428				

*Bold values indicate p-values < 0.05.*

**TABLE 4:** Panel G: Hierarchical regression models with and without predicting time in bed (TiB) adjusting for medication use.

		Unstandardized coefficients		Standardized coefficients					
Step	Predictor	B	SE	β	p	R^2^	R^2^ change	F	P
1						0.228	0.228	6.261	0.00
	Pain	618.941	765.203	0.070	0.420				
	Depression	–1651.064	533.769	–0.277	**0.003**				
	Dementia	3978.952	789.043	0.447	**0.000**				
	Age	0.48.800	51.095	0.086	0.955				
	Gender	–613.861	922.179	–0.059	0.507				
2						0.313	0.085	5.169	0.00
	Pain	511.950	740.930	0.058	0.491				
	Depression	–1611.413	525.942	–0.271	**0.003**				
	Dementia	3698.456	788.338	0.415	**0.000**				
	Age	62.678	49.355	0.110	0.207				
	Gender	–869.196	898.208	–0.084	0.335				
	Analgesics	–449.644	761.529	–0.050	0.556				
	Sedatives	–2416.144	890.838	–0.240	**0.008**				
	Antidepressants	1315.321	749.052	0.149	0.082				
	Opioids	1555.759	999.381	0.135	0.123				

*Bold values indicate p-values < 0.05.*

## Results

Mean age in the total sample was 86.2 years and 76.3% were female (see Table 1 for the number of observations for the different variables). The mean CSDD, MOBID-2, and MMSE scores were 9.5 (SD 4.7), 2.6 (SD 2.3), and 8.8 (SD 5.8), respectively. The patients from the DEP.PAIN.DEM trial had significantly higher depression and lower MMSE mean scores than the patients in the COSMOS trial (Table 2). In the total sample, 46.8% of the patients were prescribed non-opioid analgesics, 27.7% were prescribed sedative-hypnotics, 47.5% were prescribed antidepressants and 17.7% were prescribed opioids (see Table 5). Table 4 reports sleep characteristics for the full sample (Panel A), for those with and without pain (Panel B), for different levels of depression (Panel C), and for various levels of cognitive impairment (Panel D).

**TABLE 5:** Descriptive statistics for the use of analgesics, sedatives, antidepressants and opioids.

Type of medication	Percent of patients with prescriptions%	Number of observations
Analgesics (without opioids)	46.8	141
Sedatives	27.7	141
Antidepressants	47.5	141
Opioids	17.8	141

We comment on significant associations between the predictors and the dependent variables for all models that are reported in Table 4, but note that some of the regression models are not statistically significant. The hierarchical regression predicting TST showed that being in pain was associated with more TST, while more severe depression was associated with less TST (Table 4, Panel A). The regression predicting SE showed that being in pain was associated with higher SE (Table 4, Panel B). There were no significant results in the regression predicting SOL (Table 4, Panel C). The linear regression predicting WASO (Table 4, Panel D) showed that more severe dementia and pain were both associated with more WASO. The regression predicting EMA showed no significant results (Table 4, Panel E). The regression predicting DTS showed that being in pain was associated with more DTS and that higher depression scores were associated with less DTS. The final regression predicting TiB showed that more severe dementia was associated with more TiB. More severe depression was associated with less TiB (Table 4, Panel G).

In the second step of the hierarchical regressions, we adjusted for the use of analgesics, antidepressants, sedative-hypnotics and opioids. In all the regressions, the results reported above held when adjusting for the use of these medications. In addition, we noted that in the regression predicting DTS, the negative association between the use of sedatives and DTS was only marginally insignificant (*p* = 0.051) (Table 4, Panel F). Furthermore, in the regression predicting TiB, the use of sedatives was associated with less TiB (Table 4, Panel G).

In order to correct for multiple comparisons, we conducted False Discovery Rate corrections. Overall, the results largely held when taking these corrections into account. The exception was that the significant association between depression and TST became insignificant (*p* = 0.034 with a corrected threshold of *p* = 0.021).

## Discussion

Contrary to our hypotheses, NH patients with more severe pain and dementia had longer total sleep time and slept more during daytime, which indicates that NH patients with dementia and pain sleep more. Furthermore, their sleep efficiency was higher, indicating that the patients had more sleep within the time they spent in bed. In line with our hypotheses, our results showed that more severe dementia was associated with more time spent awake at night. Furthermore, people with more severe dementia spent more time in bed and people with more severe depression slept less at daytime and had less total sleep time. In addition, when adjusting for the use of medications, we found that patients who were prescribed sedative-hypnotic medications spent less time in bed.

To the best of our knowledge, this study is the first to explore how different levels of pain and depression correlate with a wide set of actigraphy-measured sleep parameters in NH patients with dementia (see Table 4). The results are of key importance since they provide new and valuable insight on the relationship between NH patients’ sleep, including sleep during daytime, and its associations with different levels of pain and depression. In light of previous research that has demonstrated that pain conditions are commonly associated with worse sleep in people without dementia (e.g., Andersen et al., 2018), the results from the present study are particularly interesting.

The results from the present study were unexpected. A potential explanation for these results could be that patients in pain receive more analgesics, antidepressants, sedative-hypnotics or opioids and thereby experience more sedation as an intended or adverse treatment effect. In turn, sedation and lack of movement could be recorded as sleep using actigraphy. However, we controlled for the use of different medications, rendering this explanation unlikely.

Another potential explanation of these somewhat surprising results may be that many older adults who experience chronic pain may develop behavior in which painful movements are avoided. This is known as the pain-avoidance effect (Vlaeyen and Linton, 2012; Achterberg et al., 2013). If this interpretation is valid, it would imply that the patients may in fact not be sleeping but are lying still in order to avoid pain, and by extension this highlight that pain assessment is fundamental. When a patient is lying still, it may be interpreted as sleep when measured with actigraphy. The fact that DTS was also higher in NH patients with pain and dementia may indicate that painful movements/activity were avoided at daytime as well. At the same time, it is noteworthy that previous research shows more agitated behavior among patients with mild to moderate pain (Husebo et al., 2014). In Blytt et al. (2018a), which is based on sleep-related data from the DEP.PAIN.DEM trial, we found that in the full sample, pain treatment improved sleep as measured with actigraphy compared to placebo after one week with pain treatment (paracetamol and buprenorphine). The results from the DEP.PAIN.DEM trial do not allow differentiation between sedation as a side-effect of the administered treatment and actual sleep. Importantly, we found no long-term effect of pain treatment on sleep (Blytt et al., 2018b). In the present study, we registered the use of different medications that can lead to sedation as a side-effect. However, this did not affect the results, and the only finding related to medication use was that the use of sedatives was associated with less time spent in bed.

Based on the present findings, we argue that there is a need for studies examining more thoroughly and systematically the association between pain, depression and sleep. It is furthermore important to acknowledge that it is very difficult to ascertain pain in people with dementia. The use of observation-based pain instruments applied by a proxy-rater who is familiar with the patients is a common method to assess pain in this population. Since a patient’s behavior is typically changed in reaction to experiencing pain, it is essential to examine pain expressions in relation to the person’s usual behavior (Hadjistavropoulos et al., 2014). Most tools used for classifying pain in NH patients are developed against this backdrop (Corbett et al., 2012; Flo et al., 2014; Lichtner et al., 2014). The MOBID-2 instrument has good validity, interrater and test-retest reliability, and responsiveness to change has been shown (Husebo et al., 2014). Yet, pain is a complex phenomenon, and we cannot be sure that the patient is experiencing pain, and in what intensity, location and duration.

Previous research indicates that increased wakefulness and fragmented sleep at night are common sleep problems in patients with dementia (McCleery et al., 2016). Our results showed that the patients with more severe dementia spent more time awake during the night. Sleep problems in patients with dementia may be a result of neural loss in the suprachiasmatic nuclei and/or other neuropathological changes to sleep-wake circuits (Swaab et al., 1985; Van Someren, 2000). The results of the present study indicate that as dementia progresses, sleep problems may be exacerbated. While more severe dementia was also associated with spending more time in bed, we cannot tell whether this is a result of the patient’s own wishes, or NH routines.

Previous studies investigating sleep and depression have found that 60 to 80% of depressed adults and older adults report having difficulty falling or staying asleep or being tired the next day (Almeida and Pfaff, 2005; Ancoli-Israel, 2009). Furthermore, depression in people with dementia is associated with lack of initiative and reduced memory, and there is thus overlap between the core symptoms of dementia and depression (Brailean et al., 2016). The present study indicates that the patients with more severe depression was somewhat more active as we found that they slept less during daytime, had lower total sleep time and spent less time in bed. A potential explanation for this could be agitation – a symptom often seen in people with dementia and depression (Husebo et al., 2014).

As pointed out by McCleery et al. (2016), there is a lack of evidence regarding drug treatment of sleep problems in people with dementia, and this is thus highly needed. However, the present study was not designed to look at the effect of sedatives on sleep in this population. Therefore, we cannot be sure if the patients receiving sedatives (27.7% of the total sample) were systematically different in other ways that can explain these results.

### Limitations

This study has several limitations. Due to the cross-sectional design of the study, we cannot draw any causal conclusions. Furthermore, the COSMOS and the DEP.PAIN.DEM trials have somewhat different inclusion and exclusion criteria. Importantly, the DEP.PAIN.DEM study, from which most of the data is derived, had depression and dementia as inclusion criteria (CSDD ≥ 8, MMSE ≤ 20). As the results from the independent samples *t*-tests showed, the patients from the DEP.PAIN.DEM trial had significantly higher depression scores and lower MMSE scores. This implies that the patients from the two trials may not be comparable, which may affect generalizability. The results from the present study may arguably be generalized to NH patients with dementia and probable symptoms of depression.

To measure sleep in the NH setting is challenging due to patients’ reduced ability to give valid self-report (Blytt et al., 2017). Therefore, objective measurement using actigraphy or use of proxy-rater assessment tools (where health personnel or relatives answer on behalf of the patient) are the current alternatives. Both have several limitations. When using actigraphy, immobility is recorded as sleep, which makes it difficult to interpret the results. Furthermore, previous research has shown that SOL has been particularly difficult to ascertain (Marino et al., 2013). When proxy-rater assessment tools are evaluated, they are often compared with actigraphy as the gold standard (Blytt et al., 2017; Hjetland et al., 2020). This indicates that actigraphy is considered a better method for measuring sleep in this population. When sleep is evaluated using proxy-rater assessment tools like the *Neuropsychiatric Inventory* and the *Cornell Scale for Depression in Dementia*, raters report significantly fewer sleep problems compared to actigraphy (Blytt et al., 2017). This highlights that measuring sleep in this population is difficult. However, individual sleep diaries – addressed by the patient’s primary nurse – would have strengthened the study.

It is furthermore important to note that we have just conducted a coarse mapping of the medications; N02A (opioids), N02B (other analgesics), N05C (hypnotics and sedatives), and N06A (antidepressants). However, effects such as sedation may differ within the different drug groups and according to the prescribed dose. Additionally, we did not assess whether the daily prescriptions were in fact administered to the patient. Only daily prescribed treatments were recorded we did not control for the use of as-needed treatment.

Another limitation in the present study was that we did not have complete datasets for all the different variables (for an overview of missing data, see the number of observations in Table 1). We cannot be sure whether the results would be different if we had complete data for all the relevant parameters investigated.

Together, the results of the present study revealed new and interesting insights on the relationship between sleep, pain and depression in a fragile patient group. Importantly, the results indicated that being in pain was associated with more sleep as measured with actigraphy, also when controlling for use of potentially sleep-enhancing medications like sedative-hypnotics, analgesics, opioids and antidepressants. Future research should focus on further investigating the mechanisms underlying these results.

## Data Availability Statement

The datasets generated for this study will not be made publicly available in order to maintain confidentiality of the study participants. Requests to access the datasets should be directed to the corresponding author.

## Ethics Statement

The studies involving human participants were reviewed and approved by The Regional Ethics Committee REC-West 2013/1474 and REC-West 2013/1765. The patients/participants provided their written informed consent to participate in this study.

## Author Contributions

BH is the primary investigator of the COSMOS and the DEP.PAIN.DEM trial from which the data originates. KB designed the study, analyzed the data, and wrote the manuscript. BB, AE, EF-G and BH designed the study, helped with the analysis of the data, and wrote the manuscript. All authors have read and approved the manuscript prior to publication.

## Conflict of Interest

The authors declare that the research was conducted in the absence of any commercial or financial relationships that could be construed as a potential conflict of interest.
